# Recent Advances and Perspective of Nanotechnology-Based Implants for Orthopedic Applications

**DOI:** 10.3389/fbioe.2022.878257

**Published:** 2022-04-25

**Authors:** Ming-qi Chen

**Affiliations:** Traumatic Orthopedics Yantai Mountain Hospital, Yantai, China

**Keywords:** nanotechnology, orthopedic, 3D implants, pores architectures, nanotechnology-driven biomaterials

## Abstract

Bioimplant engineering strives to provide biological replacements for regenerating, retaining, or modifying injured tissues and/or organ function. Modern advanced material technology breakthroughs have aided in diversifying ingredients used in orthopaedic implant applications. As such, nanoparticles may mimic the surface features of real tissues, particularly in terms of wettability, topography, chemistry, and energy. Additionally, the new features of nanoparticles support their usage in enhancing the development of various tissues. The current study establishes the groundwork for nanotechnology-driven biomaterials by elucidating key design issues that affect the success or failure of an orthopaedic implant, its antibacterial/antimicrobial activity, response to cell attachment propagation, and differentiation. The possible use of nanoparticles (in the form of nanosized surface or a usable nanocoating applied to the implant’s surface) can solve a number of problems (i.e., bacterial adhesion and corrosion resilience) associated with conventional metallic or non-metallic implants, particularly when implant techniques are optimised. Orthopaedic biomaterials’ prospects (i.e., pores architectures, 3D implants, and smart biomaterials) are intriguing in achieving desired implant characteristics and structure exhibiting stimuli-responsive attitude. The primary barriers to commercialization of nanotechnology-based composites are ultimately discussed, therefore assisting in overcoming the constraints in relation to certain pre-existing orthopaedic biomaterials, critical factors such as quality, implant life, treatment cost, and pain alleviation.

## Introduction

Nanotechnology is a multifaceted area where the physical, chemical, and biological characteristics and materials’ structures are altered at the nanoscale scale. Nanomaterials exhibit new size-dependent characteristics that are not often seen in bulk materials. Nanotechnology advancements have paved the way for a plethora of novel uses in medicine (Kaur et al., 2015; Rani et al., 2015; Liu et al., 2019), molecular biology (Ulijn and Jerala, 2018; Kumar et al., 2019a), biotechnology (Rani et al., 2018), and environmental research (Bhanjana et al., 2019a; Kumar et al., 2019b; Kumar et al., 2019c). Nanotechnology has been applied to the drug (e.g., nanomedicine) with the development of a number of approaches for the diagnosis, prevention, and management of a variety of ailments, for instance tumour treatment, tissue engineering (TE) scaffolds, imaging in medicine, medicine distribution, and immunotherapy. Nanomaterials are very intriguing prospects for the fabrication of future orthopaedic grafts because of their capacity to imitate or recreate the component organs of natural bone (Wang et al., 2016; Cheng et al., 2018). In orthopaedic management, bone replacements are required to repair irreparable damage to native, healthy bone. Nanomaterials may contribute significantly in this regard by giving cellular structural support (for example, scaffolds with nanofunctionalization) and influencing cell propagation, migration, and differentiation (Patel et al., 2016; Vieira et al., 2017).

**FIGURE 1:**
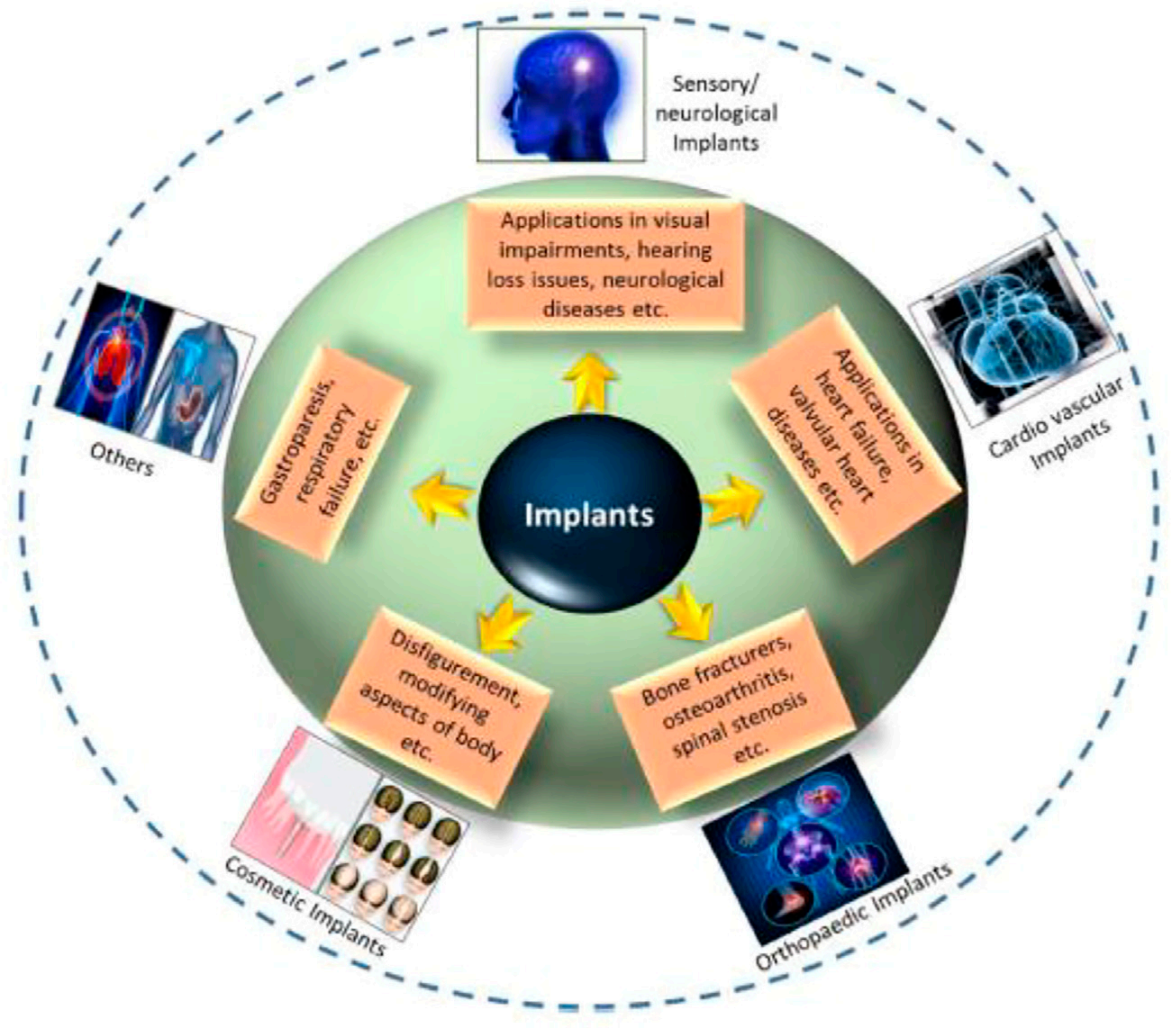
Application-oriented categorization of bioimplants into categories such as cardiovascular grafts, aesthetic grafts, neurological/sensory grafts, orthopaedic implants, and other applications is provided. Reproduced with permission from (Kumar et al., 2020).

The bioimplant industry is rising exponentially as the population ages, lifestyle changes (particularly those that encourage and prolong chronic illnesses for instance, cardiovascular and osteoarthritis disease, technical improvements in bioengineering, and improved cosmetic grafts awareness, all contribute to the growth of the bioimplant market. According to marketplace research studies, the worldwide market for bioimplants is expected to reach $115.8 billion by 2020, expanding at a 10.3% compound annual growth rate (CAGR) throughout the forecast period (2014–2020) (Zhao et al., 2021). Bioimplants have developed as a potentially transformative treatment option for visual impairments, neurological illnesses, orthopaedic problems, cardiovascular disease, deformity, and dental abnormalities (Figure 1) (Ghezzi et al., 2013; Jackson, 2016; Scaini and Ballerini, 2018). Numerous bioimplants, including joint replacements, bone plates, heart valves, vascular grafts, sutures, dental grafts, ligaments, and intraocular lenses, are frequently used to change or restore function to destroyed or disintegrating tissues, 2) adjust the functions of a physical component, 3) help in curing, and 4) repair abnormalities for cosmetic reasons (Cross et al., 2016). Numerous engineering approaches have been described that use common metallic/non-metallic materials to imitate the physical qualities, chemical properties as well as organ or tissue gradient architecture. However, typical bioimplants have significant limitations. They infrequently react to tissues and are incompatible with tissues, and the human body does not always accept them (Bian et al., 2016). Nanotechnology’s effect on the implant sector has accelerated in recent years. Nanomaterials with biologically inspired properties, in particular, are prompting researchers to investigate their potential in improving the function of traditional implants. This article discusses the evolution of biomaterials in orthopaedic applications, from traditional (e.g., metallic and non-metallic) resources to nanoparticles. Orthopaedic treatments rely heavily on an accurate diagnosis of curative sites and efficient embedding. To present a complete overview of this rapidly expanding scientific field, current developments in important orthopaedic biomaterials like smart biomaterials, porous materials, 3D printable nanocomposite grafts are covered and commercial problems. This study presents a solid foundation for incorporating orthopaedic grafts powered by nanotechnology into the human body.

## Drug Delivery Using Nanotechnology Implantable Materials

Though the primary emphasis of nanophase drug delivery devices has been on treating and preventing infections associated with implants and prosthetic joints, this novel method has also been shown to be effective in cancer diagnosis and treatment, as it allows for a more targeted assault on cancer cells and can also in the development of bone when combined with anabolic drugs to reduce osteolysis about surfaces of prosthetic joints (Puckett et al., 2010). A new exciting field of study is the production of injectable pharmaceuticals encapsulated in nanospheres capable of prolonging a drug’s pharmacological impact. Some of them are explained below.

### The Role of Nanotechnology-Based Implants in Musculoskeletal System and Oncology

Nanotechnology-based implants in orthopaedic oncology can significantly enhance diagnostics, overcome medication resistance, reduce peripheral damage to usual host tissue, and more efficiently provide pharmaceuticals to tumour cells (Savvidou et al., 2016). Nanomaterials are capable of carrying ligands. By incorporating precise ligands that bind to the distinct genes produced by cancer cells can increase the capacity to diagnose primary and metastatic malignant bone cancers early and precisely. By incorporating contrast agents into the nanoparticles, it is possible to improve the accuracy of targeted tumour imaging and to determine tumour survivability, which might be highly useful for clinical evaluation and surgery planning. Tumour cells increase resistance by producing MDR (multidrug resistance proteins) on their surface, which acts as a pump, removing the tumour drug from the cells and lowering its plasma concentrations. Nanoparticles enable the development of vehicles capable of efficiently delivering cancer treatments into cells but also delivering certain genetic codes capable of bypassing MDR proteins. Nanophasic drug carriers techniques enhance tumour cell targeting both actively and passively. Following endocytosis, drug-loaded nanomaterials may be conjugated with a polymeric material such as folic acid and mannose to determine the target tumour cell. Additionally, nanomaterials enable larger medication concentrations within cancer cells because of their reduced size (passive targeting) and the permeability of tumour cells. The growth of progenitor cells and hematological stem using zwitterionic hydrogels might assist the practical use of hematopoietic stem cell therapy (Bai et al., 2019).

Orthopedic implants are often placed in individuals who have had bone cancer resections. However, conventional materials are not intended to suppress cancer development or recurrence. As a result, attempts are being made to develop implants that promote normal bone formation whereas inhibiting tumor growth. Selenium has previously been proven to possess similar qualities, and nano-selenium grafts have been shown to prevent malignant osteoblast proliferation while supporting good osteoblast function at the implant-tissue line (Tran et al., 2010). Unlike untreated titanium implants, it was discovered that the selenium nanomaterial improved calcium accumulation, bone adherence, bone development, and activity of alkaline phosphatase. In recent times, nanosized magnesium alloy grafts with grain refinement revealed anti-tumor effects. Human osteosarcoma cells were less survive and adherent to this substance (Nayak et al., 2016).

### Use of Nanotechnology-Based Implants in the Treatment of Osseous and Chondral Abnormalities

Handling segmented bone abnormalities caused by failed fixations, trauma, or arthroplasty poses a significant difficulty. Present methods of correcting these problems, including auto/allografts and metal matrix replacement, use their drawbacks, including limited supply, infection risk, and inadequate scaffolding qualities, which restrict the amount of osteointegration. Given that the optimal scaffold for osteointegration is determined by the degree of contact between the biomaterial and the host tissues, nanostructured nanomaterials are excellent because they may be colonized by bone cells (Andreacchio et al., 2018). The ideal frameworks must be recyclable and operate as an extracellular matrix upon which cells may act together, multiply, and differentiate into natural tissues.

Nanomaterials polymers are capable of providing structural support and pore sizes that are appropriate for cell migration and activity, as well as acting as a medium for cell movement and activity. Additionally, they may give biochemical markers by including growth factors and chemokines to regulate tissue conversion, as well as mechanical support by delivering peptide arrangements that bind receptors and trigger intracellular signalling pathways. These characteristics of nanoparticles make them suited for treating major bone defects (Luthringer et al., 2013). Once their structural, biological, biochemical, and templating activities are complete, nanoscaffolds will resorb, permitting for a further natural restoration deprived of the complications related to implants and biomaterials that are not disintegrable (Roddy et al., 2018).

Numerous nanostructured materials, both natural and manmade, have been investigated to treat bone abnormalities. While natural nanomaterials provide high biocompatibility, they have improper handling properties and insufficient structural support. On the opposite hand, synthetic materials give superior structural support but are not biocompatible. Synthesized nanomaterials, like bioactive ceramics hydroxyapatite (HA) and (tricalcium phosphate (TCP) and derived products), composites like poly-lactic acid (PLA), poly-glycolic acid (PGA) and polymeric matrix, are presently recommended as scaffolding substances for managing osseous deformities due to its capability supply enhanced structural strength. External treatment with growth factors for example bone sialoproteins (BSP) and bone morphogenic proteins (BMP) might enhance the capacity of these nanostructured biomaterials to attain effective osteointegration. Gelatin and fibrin, two natural polymers, have been employed to repair bone lesions in non-weight-bearing locations such as cranium deformities.

Cartilage has a more complicated structure, making it more challenging to cure cartilaginous abnormalities using synthetic or biological scaffolds. Due to their superior biocompatibility, cell infiltration, biodegradability and neovascularization, scaffolds made of biological proteins such as polysaccharides and collagen scaffolds such as chondroitin sulphate, hyaluronic acid, agarose and chitosan are chosen for repairing cartilage abnormalities (Vasita and Katti, 2006). Despite its immunogenicity, type I collagen scaffolds are the most preferred. In patients with chondral abnormalities, acid-treated collagen gels containing mesenchymal stem cells have been demonstrated to generate hyaline-like cartilage. Gelatin is a denatured collagen substitute that does not cause immunoreactivity or disease transmission.

Given that most cartilage lesions are treatable to minimally invasive surgical methods, the accessibility of injectable scaffolds becomes critical. Hydrogels are nanoscale networks made of polymers composed of gelatin or collagen that are injectable and can harden and conform to the shape of the defect after implantation. Hydrogels have been demonstrated to form a cartilage-like extracellular matrix with gradual enhancement in mechanical characteristics owing to the persistent accumulation of glycosaminoglycan-rich matrix when equipped with chondrocytes and injected.

The use of nanofibers to fabricate chondrogenic or osteogenic scaffolds has presented a number of benefits, including increased cell attachment, migration and multiplication. The nanotube scaffolds included the greatest proportion of collagen type II, an increased capacity to adsorb human blood proteins, and a considerable important overexpression of cartilage-explicit proteins and genes, for example collagen II and IX. Numerous available studies have revealed that cartilage and bone tissue engineering abnormalities is one of the most significant uses of nanotechnology and associated investigation in orthopaedics (D'Antimo et al., 2017).

## Classification of Orthopaedic Biomaterials and the Issues

**FIGURE 2:**
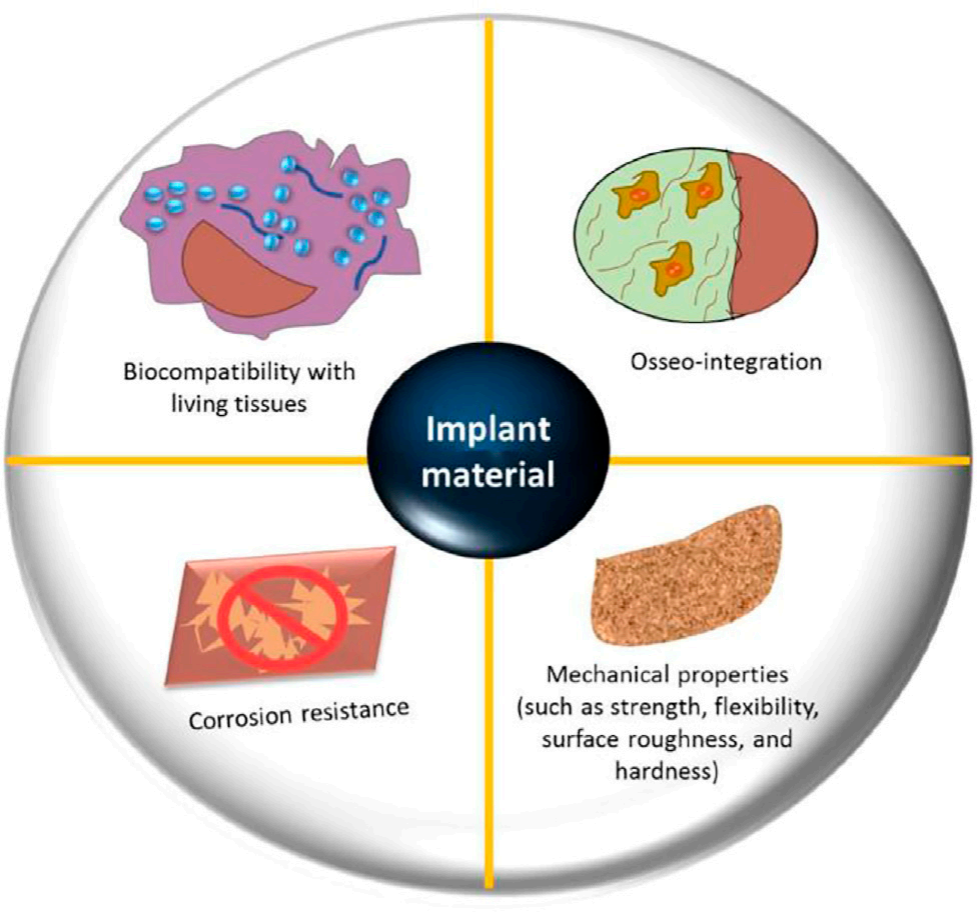
Important design parameters for orthopedic biomaterials include biocompatibility with live tissues, osseointegration, resistance to corrosion, and mechanical qualities (flexibility, strength, roughness, surface and hardness). Reproduced with permission from (Kumar et al., 2020).

Biological tissues are divided into two broad groups: soft tissues (which include skin, fibrous tissues, synovial membranes, and ligaments) and hard tissues (which include cartilage, bone, nails and teeth), which may or may not have mineral elements. The scarcity of donor organs prompted researchers to develop innovative methods for simulating or replicating organs (Carvalho et al., 2018). Bioimplants have been developed to repair, sustain, or enhance the functionality of human tissues in response to this need. While biomaterials designed for graft applications are similar to biomolecules like tissue and bones, they are not identical. These biomaterials may be synthetic or natural in origin and are designed to function properly in a biological setting. In orthopedic implants, biomaterials are used to either restore the structural stability of broken bone or to change it completely. Each biomaterial must meet several critical requirements, including appropriate mechanical possessions (i.e., elastic modulus and precise weight), Good biostability (Hydrolysis, corrosion, and oxidation resistance), biocompatibility, in the instance of bone prostheses (osseo-integration), high bio-inertness (non-toxic and non-irritant), high wear resistance, and ease of operational application (Figure 2) (Mitragotri and Lahann, 2009).

Biological biomaterials have been presented to promote cell propagation and tissue remodelling (Sridharan et al., 2015). Important investigating efforts have been done throughout time to develop and manage biomaterial qualities to achieve application-specific biological responses (Huebsch and Mooney, 2009; Achterberg et al., 2014). For example, by varying the stiffness of the cell-substrate, it is feasible to maximize muscle cell development. Orthopaedic biomaterials may be grouped into two broad types in the current era: Conventional biopolymers and nanostructured biomaterials. The conventional biomaterials may be broadly categorized into the following categories: 1) materials that are nonmetallic (for example amorphous glasses, polymeric, carbon composites and ceramics with crystalline structure) and (ii). metals and their alloys. This paper discusses traditional biomaterials and the issues connected with their use in orthopaedic implant applications.

### Alloy and Metals

Metals and alloys are often selected for load-bearing and anterior fixing orthopaedic grafts. These grafts are firmly attached to bones, ensuring minimum mobility between the implant and the host tissue, along with load-bearing capability at the insertion site. While these materials are widely available, Only a fewer are biodegradable, and therefore competent of long-term achievement in graft applications, for example, magnesium in recyclable orthopaedic grafts for load-bearing (Julmi et al., 2019), stainless steel surgical grade (normally 316 L) in impermanent grafts (for example, fracture plates and hip nails) (Zlotnik et al., 2019), the use of titanium in joint and bone restoration (Kaur and Singh, 2019; Zhang and Chen, 2019), orthopaedic prosthesis, as well as cobalt-based alloys (for shoulder, hip, and knee) (Bandyopadhyay et al., 2019). Specific mechanical qualities are needed in orthopaedic biomaterials applications for I stabilising or stimulating fracture integrity, 2) realigning bone fragments, and 3) joint replacements. Metallic nanomaterials were originally employed in the 1860s, during the industrial revolution, when the metal industry was expanding (Lausmaa et al., 1990). Due to their homogeneous qualities (for instance, high toughness, tensile strength, and durability), simplicity of fabrication, and appropriate biocompatibility, metallic materials play a crucial role in biomedical implant engineering, which are all desired for implant lifetime (Kaur et al., 2017; Jenko et al., 2018; Dogra et al., 2019). Titanium (Ti) and its alloy Ti6Al4V are popular bioimplant ingredients with fatigue performance, corrosion resistance, great biocompatibility, reduced cobalt concentration, and lightweight. The zwitterionic metal-chelating polymers exhibit enhanced biodistribution, increased blood levels of radioactivity, decreased absorption by normal tissue, and increased tumour uptake. Using the extracellular domain of HER2, surface plasmon resonance investigations examined that the MCP immune conjugates retain excellent antigen binding, with dissociation constants in the low nM range (Liu et al., 2015). Using a fluorescein isothiocyanate/neutravidin receptor as an intermediate, a biotin end group enabled conjugation to biotinylated beads. To demonstrate the material’s behaviour, high-quality image mass cytometry studies based on^115^ while identification was carried out. The findings establish a possible future use for mass cytometry of the [In(cb-te2pa)]+ complex-based polymers for bio-implants (Grenier et al., 2020).

**FIGURE 3:**
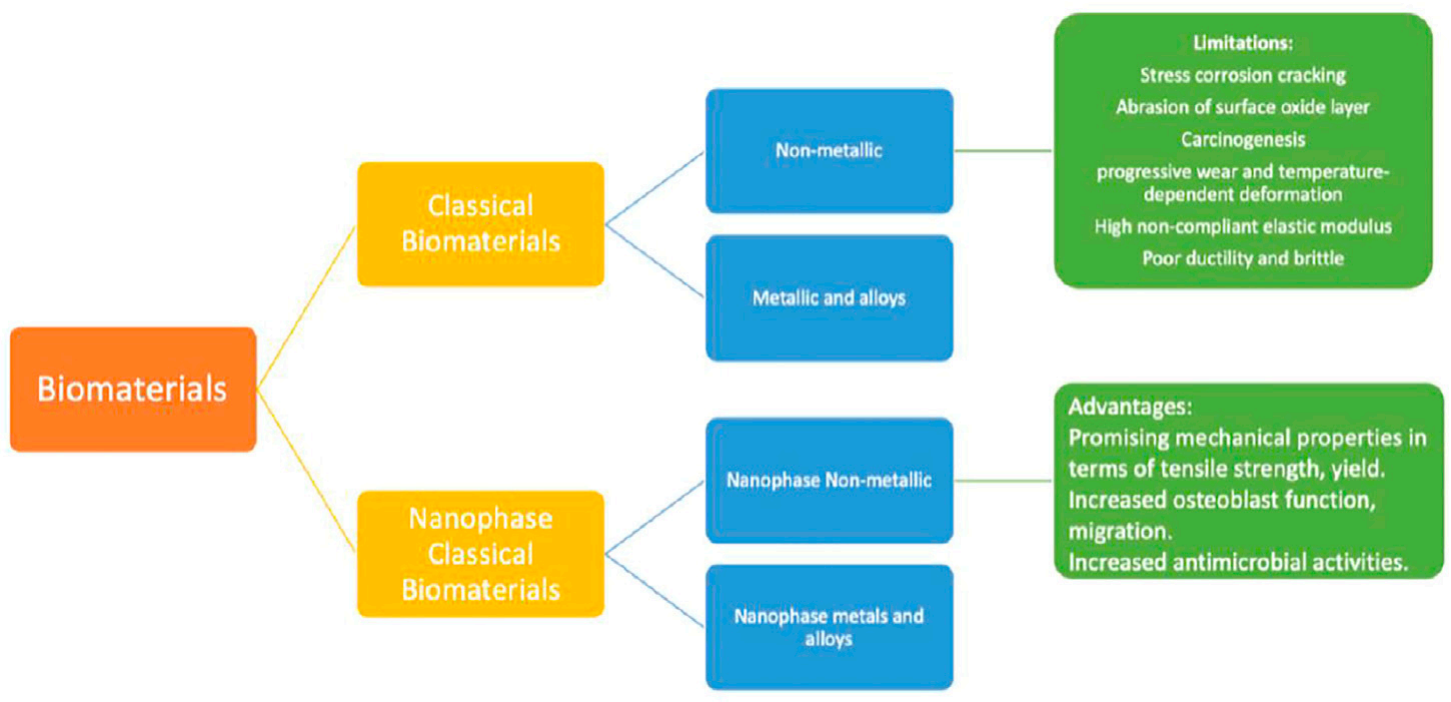
Orthopaedic biomaterials are classified into two broad groups: conventional and nanophase biomaterials. The earlier is more classified into (1) metallic and alloyed biomaterials and (2) non-metallic biomaterials. In the same way, the latter may be categorized into I nanophase metallic (and alloyed) biomaterials and (3) non-metallic biomaterials in nano-phase. Numerous disadvantages exist for conventional biomaterials in implant applications, including corrosion process (in the event of 316 L steel material), gradual wear, and thermally distortion are some of the problems that might occur (in the incident of polymers). In comparison, nanoparticles exhibit many desirable qualities, including increased osteoblast functions and superior mechanical belongings (in terms of yield strength and tensile, etc.).

Developing a coating of oxide on a metal surfaces provides great corrosion resistance. The oxide coating constancy has been identified as a significant concern with bioimplants, as it may alter as a result of interactions between the live tissues and metallic surface (Kurella and Dahotre, 2005). The majority of metallic elements dissolve chemically and/or electrochemically in the human body’s environment, which contains an oxygenated saline solution with a 0.9 percent salt content at a pH of 7.4 and a temperature of 37°C (Figure 3) (Manrique-Moreno et al., 2011). n this oxygenated salt solution, including living environment, in solution, such metallic nanomaterial have a potential to lose electrons. As a consequence, these biomaterials are prone to corrode in such environments, which may result in inflammation and implant loosening (Li et al., 2003; Manam et al., 2017). The complicated combination of the biological environment’s corrosive properties and physiological stressors may result in the early failure of metallic bioimplants. Stress corrosion cracking of recyclable metal grafts is the reason of this early failure, which may result in considerable material degradation and limit the implant’s life duration (Choudhary et al., 2014). In orthopaedic implants, 316 L stainless steel has been documented to fail permanently owing to its low fatigue forte and/or susceptibility to plastic deformation (Reclaru et al., 2001).

### Non-metallic Materials

Numerous non-metallic substances have sophisticated structural implantation characteristics, including, crystalline ceramics, carbon composites, polymers, and amorphous glasses. Due to their intrinsic bioincompatibility or lack of mechanical qualities, these materials were not widely used. Significant effort has been made to develop them as structural grafts (Denry and Kelly, 2014). Polymeric substances are favored for 1) tissue engineering with porous scaffolds (owing to their increased osseointegration and bone regeneration capabilities) and 2) regulated drug biocompatibility and excellent electromechanical characteristics) (Middleton and Tipton, 2000; Farah et al., 2016). In contrast to metallic grafts, polymers may progressively transmit stress to a damaged location, allowing for optimal tissue healing (Ramakrishna et al., 2001), and 2) slowly restoring tissue function without enzymes or catalysts. At first, non-reinforced recyclable polymers demonstrated 36% more tension and 54% greater bending than strengthened steel materials. Fiber reinforcement may increase the strength of polymeric materials; 62 percent and 15% stiffness (in contrast to stainless steel) can be attained by polymer reinforcement with non-biodegradable carbon fibre and biodegradable inorganic fibre, correspondingly (Daniels et al., 1990). Prior to implanting devices into the human body, it is critical to cautiously pick the packaging material that will connect the graft instrument to the human body. These substances must be able to stop waste materials from transferring between them. Poly (lactic acid) (PLA), polyhydroxyalkanoates (PHAs), ultra-high molecular weight polyethylene (UHMWPE), polyvinylidine fluoride (PVDF), polyglycolide (PGA), and polyether ether ketone (PEEK) are the most often utilised polymers for packaging orthopaedic implants (Athanasiou et al., 1998; Nair and Laurencin, 2007). Advanced wear and temperature-dependent distortion under loading are the primary problems with polymers, which is equivalent to corrosion in the case of metallic grafts (Sabir et al., 2009). The most prevalent issue with UHMWPe is oxidative deterioration throughout shelf life, which may be improved further (for instance, by certain suitable crosslinking procedures). However, because of their low friction constant with metal, they seem to be more encouraging as a behaviour surface in complete joint expedients (Sobieraj and Rimnac, 2009).

## Orthopaedic Implants Using Nanotechnology

Nanomaterials have previously been studied for bioimplant properties because of its bioactive character and programmable surface possessions. Nanomaterials with larger surface area, efficient rigidity, smoothness, and changed physical and chemical properties enhance 1) adherence, 2) propagation, 3) bone-related protein synthesis, and 4) calcium containing mineral accumulation (Sobieraj and Rimnac, 2009; Waterman et al., 2011; Xu et al., 2011). Nanomaterials can be used in orthopaedic grafts since they can decrease the ratios of biological bone’s essential components. For instance, nanomaterial monomers and nanocomposites have been extensively studied in bone tissue engineering to stimulate osteoblast activity, improve osteointegration, and repair bone-related disorders (Rezwan et al., 2006). The nanosized used in implants, such as nanopillars (Bhanjana et al., 2017a), nanotubes (Kumar et al., 2018), nanocubes (Bhanjana et al., 2016), quantum dots (Bhanjana et al., 2019b), nanorods (Clifford et al., 2019), nanoflowers (Bhanjana et al., 2017b), and metal-organic frameworks (Nehra et al., 2019), are critical to consider. Numerous research has been conducted to investigate the beneficial surface features of nanoscale ingredients that might encourage or enable 1) a high number of precise protein relations, 2) improved osteoblast increment, and 3) improved osteoblast development and movement for more proficient bone growth than traditional tools (Zhang, 2004; Tran et al., 2009).

### Nanomaterials Implantation

With the development of nanotechnology, a wide range of nanophase (100 nm particle size) components, comprising ceramics, metals, composites and polymers have been developed with unique surface characteristics; several of these materials display an improved capacity for osseo-integration and new bone development (Zhang and Webster, 2009). The decline of titanium particle size from 4,500 nm to 200 nm (manufactured by similar channel angular pushing) was claimed to boost cell propagation by a factor of 20 (Estrin et al., 2009). Nanophase ingredients have a high density of grain boundaries due to their different atomic structure. Nanocrystalline materials, a polycrystalline material having crystallites as small as a few nanometers in diameter, provide excellent strength and/or hardness but are brittle and/or ductile (Koch, 2003; Meyers et al., 2006). It is worth noting that the inelastic character of nanoscale materials might provide insurmountable challenges in advanced structural applications. Grain boundaries are predicted to have a significant role in the thermodynamic (Kirchheim et al., 1988) and kinetic characteristics of hydrogen in metals and hydride production (Mütschele and Kirchheim, 1987a). Historically, this occurrence has been related to hydrogen embrittlement, hydrogen storage, and metal hydrides as hydrogen sensors (Wadell et al., 2014). In recent times, the function of lattice coherency strain and displacement nucleation in particle-size dependence of hydride production has been examined (Mütschele and Kirchheim, 1987b; Griessen et al., 2016) for single crystal nanoparticles.

**FIGURE 4:**
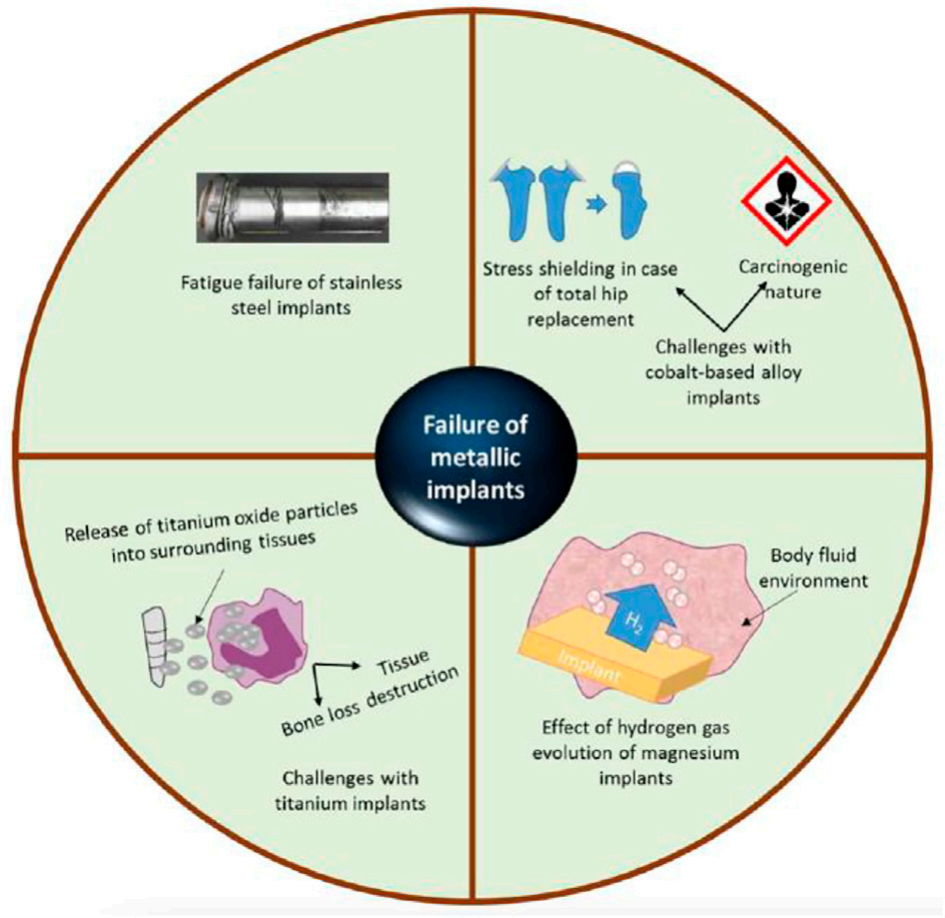
These problems are linked with traditional metal grafted orthopaedics: I stainless-steel graft fatigue failure (i2) cobalt alloys’ stress protection and carcinogenicity (i3) titanium grafts release oxide particles into the nearby tissues, which can induce tissue damage or bone loss, and (i4) the impact of hydrogen gas evolvement on magnesium orthopaedic grafts. Reproduced with permission from (Kumar et al., 2020).

Numerous factors contribute to the brittle character of nanostructured materials, including their compact manufacturing and basic nature (Zhao et al., 2006). Typical nanostructures were observed in orthopaedic implants (Webster and Ejiofor, 2004; Puckett et al., 2010; Zhou and Lee, 2011; Costa et al., 2012; Misra et al., 2013). Zhang et al.(Zhang et al., 2013) discovered enhanced mechanical characteristics (i.e., 31.7 GPa hardness and 314 GPa Young’s modulus) in nanomaterial MgAl2O4 ceramics (40 nm grain size) manufactured via sintering at high pressures and temperatures. Serra et al. (Serra et al., 2013) developed a nanostructured Ti6Al4V alloy by subjecting pure titanium to extreme plastic deformation. The nanostructured Ti6Al4V alloy displayed superior mechanical characteristics than pure titanium, containing an 1,240 MPa ultimate tensile strength against 700 MPa 2) 1,200 MPa yield stress against 530 MPa yield stress, and 3) 12 percent elongation against 25 percent for pure titanium. Despite the above mechanical characteristics, nanostructured materials roughness had a significant effect on osteoblast function; Surface texture virtues for standard titanium and 03 nanoscale ingredients (Ti6Al4V, Ti, and CoCrMo alloy) were 4.9, 11.9, 15.2, and 356.7 nm, correspondingly. Improved functions of osteoblast have been shown using various nanoscale materials (for example, Ti6Al4V, Ti, and CoCrMo) in conjunction with decreased competitive cell functions (Liu et al., 2014). Increased osteoblast proliferation (measured in cells/square cm after 5 days) was reported with all nanophase materials, containing titania (8000), alumina (6000), and HA (9000), as compared to standard borosilicate glass (5000) (Webster et al., 2000). Figure 4 decipts the various reasons responsible for the failure of metalic implants.

### Diagnostic Applications

Nanotechnology’s based implant function in cancer detection is predicated on the ability of nanomaterials complexes to attach to particular genetic alterations, allowing for comprehensive imaging on a cellular scale. By adding a counter-argument to these combinations, tumour cells expressing the precise mutation may be seen (Savvidou et al., 2016).

Unlike MRI, there are several detecting tools available that use nanotechnology and are altering the area of orthopaedics. For example, in the case of osteoporosis, diagnostic procedures are critical in delivering exact data finding in a fast, inexpensive, and non-invasive way. Earlier to the invention of nanomaterials-based approaches, there were few viable detection methods (Garimella and Eltorai, 2017). Nevertheless, emerging nanotechnology-based technologies enable the diagnosis of osteoporosis utilising a portable device. Notably, study has resulted in the creation of a unique biochip that employs gold nanoparticles to detect an osteoporosis-associated protein. It has been shown to assess bone quality successfully and to accurately detect and identify the amount of bone deterioration (Garimella and Eltorai, 2016; Garimella and Eltorai, 2017). Additionally, utilising fluorescent probes to identify nanoparticles may assist in the evaluation of cancer response to treatment (Hennig et al., 2015). This approach may provide a more precise residual tumor volume estimate than histologic examination after tumour removal (Young et al., 2012).

## Arthroplasty

### Material for the Implant

**FIGURE 5:**
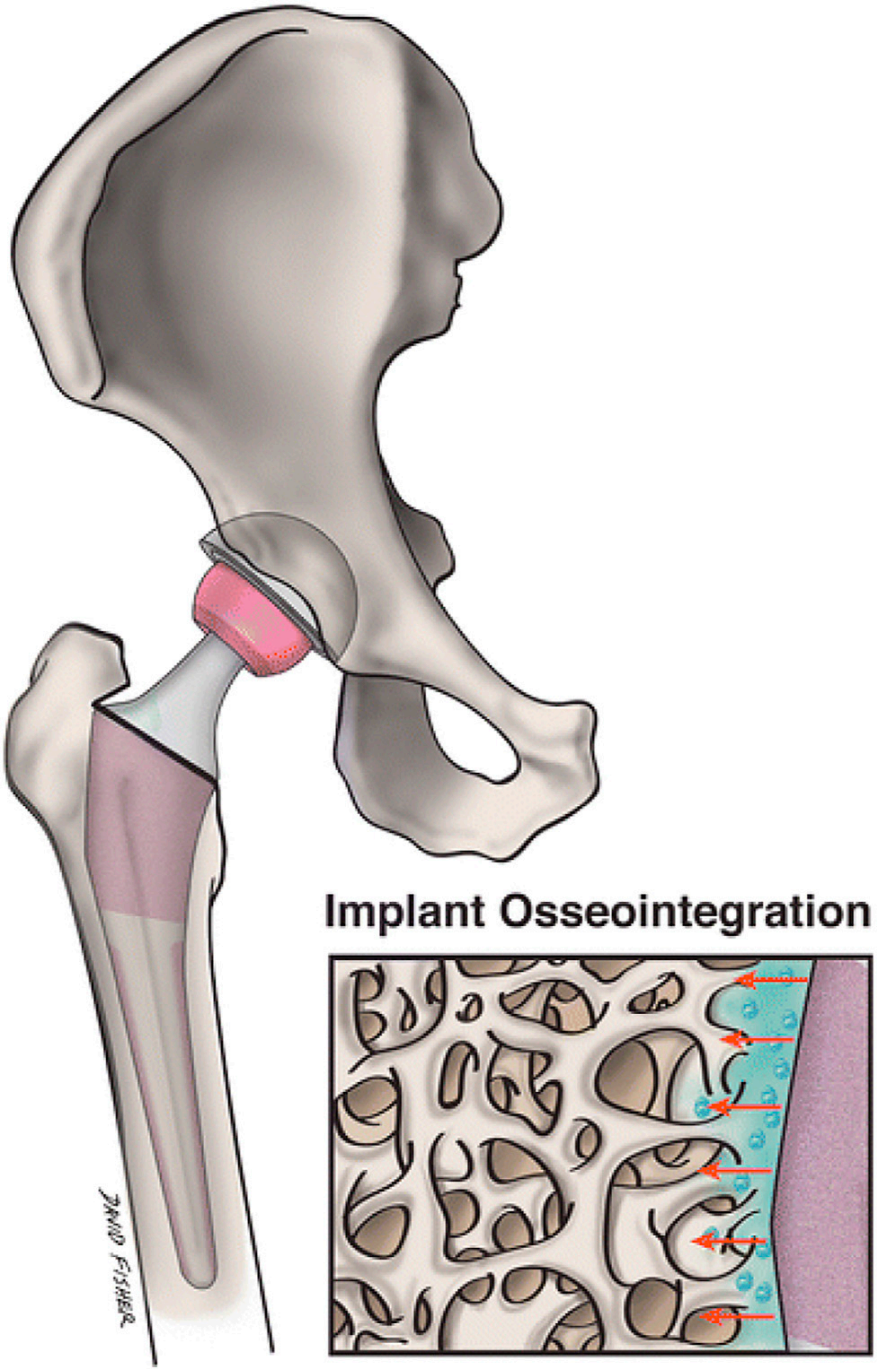
Nanostructured grafts could more closely replicate the environment of natural bone and promote transplant osseointegration and nearby osteogenesis more than conventional implants. A magnified image of a nanoengineered graft surface and its geographical interaction with neighbouring bone is shown in this figure. Reproduced with permission from (Smith et al., 2018).

When it comes to main joint replacement, it is a very effective procedure, its lifetime is limited. In arthroplasty, nanotechnology concentrates on creating implantable substances that are safe and effective while also prolonging the average lifetime of grafts and reducing infection. By modifying key surface properties of the implant, a more favourable contact between the surrounding and the implant bone may be produced (Figure 5). Nanotextured graft surfaces have been shown to enhance osteoblast activity and development, hence increasing graft osseointegration (Gusić et al., 2014). Especially, the method of severe plastic deformation (SPD) has shown the capacity to enhance titanium graft biocompatibility and mechanical properties by degrading the metal granules ranging from coarse to nanoscale when exposed to a complicated high-stress condition (Serra et al., 2013). Due to concerns about possible fracture, the use of UHMWPE inserts in arthroplasty has been restricted. However, because to its wear resistance and superior biocompatibility, attention in increasing the mechanical stability of UHMWPE by nanotechnology has grown. The incorporation of carbon nanotube into this polymer has proven translational effectiveness and could sometime be used as an acetabular liner or component of the tibia (Puértolas and Kurtz, 2014). By modifying the nanostructure of a transplant’s surface, it is possible to strengthen its confrontation to static and dynamic tiredness, develop functioning, and promote implant survival.

### Medicine in Sports

#### Chondrogenesis

**FIGURE 6:**
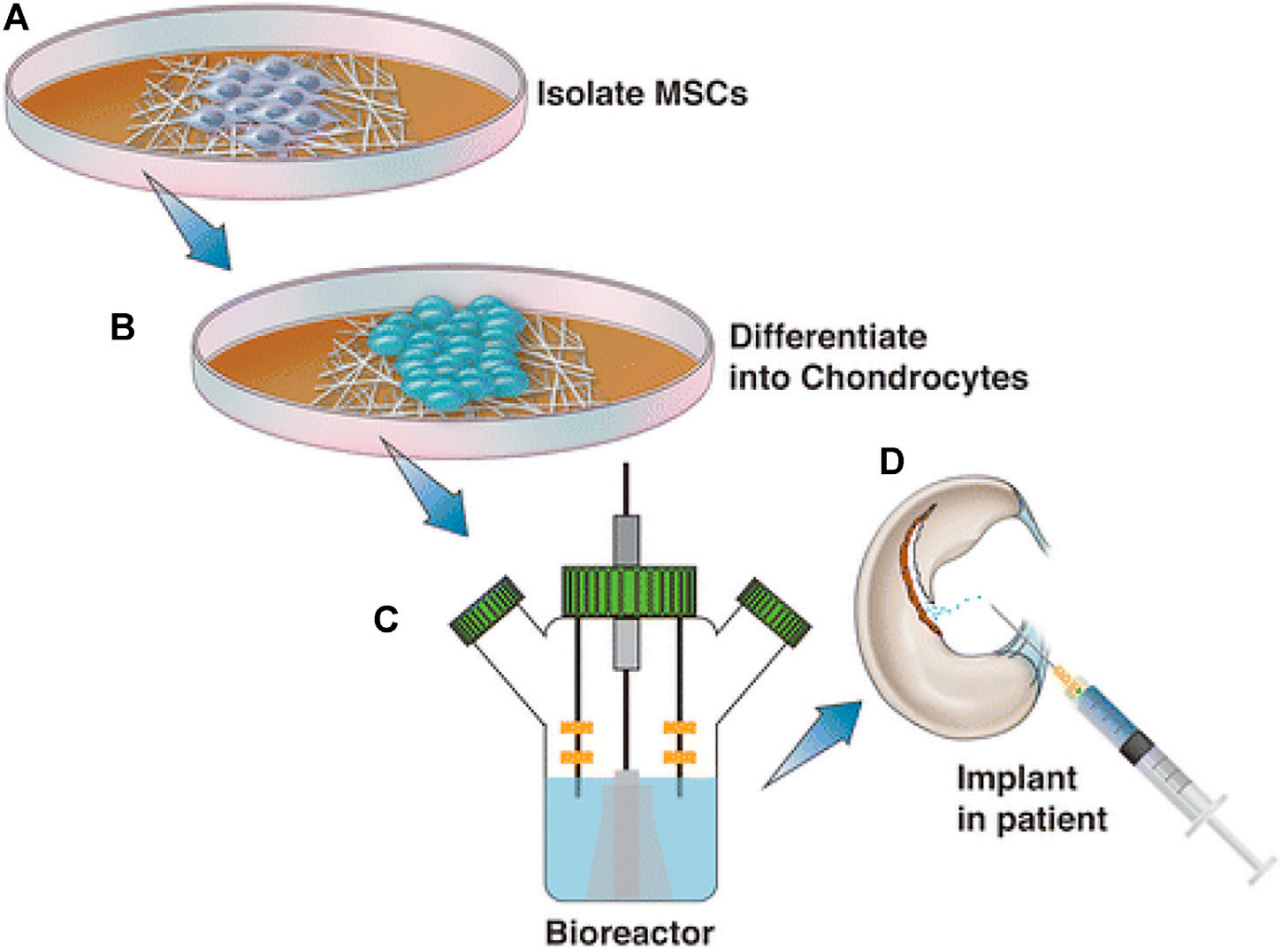
Although regenerative approaches using human MSCs have had little success in treating osteochondral abnormalities, nanotechnology could improve the effectiveness of these treatments. This graphic depicts the normal sequence of events during the treatment of MSCs with nanotechnology. The patient’s MSCs are initially isolated and cultivated in a growth medium **(A)**. After differentiation into chondrocytes **(B)**, such cells are embedded into the chosen material for scaffolding, grown in a bioreactor, and then reinserted into the patient **(C)** and **(D)**. Reproduced with permission from (Smith et al., 2018).

The healing of cartilage blemishes has been a subject of intense study in the area of biomedical sector. Fully developed cartilage tissue lacks the appropriate healing response for full redevelopment, which results in gradual deterioration leading to osteoarthritis if left untreated. Preclinical attempts to increase MSC treatment (Figure 5) via emerging a biocompatible scaffold that facilitates instinctive cartilage healing have met with primary achievement (Mahboudi et al., 2018; Ustun Yaylaci et al., 2016; Liu et al., 2014). Yaylaci et al. (Ustun Yaylaci et al., 2016) used nanofibers to create a hyaluronic acid analogue that promoted MSC development into the correct chondrogenic lineage without the toxicity associated with natural scaffolds. Liu et al.(Liu et al., 2014) used pluripotent stem cells to create a nanocomposites scaffold made of gelatin and polycaprolactone that accelerated osteochondral regeneration and subchondral bone repair. Mahboudi et al. recently reported that using a nanofiber-based polyethersulfone scaffold dramatically increased MSC chondrogenesis (Mahboudi et al., 2018). Apart from the aforementioned research, a range of alternative scaffolds for treating cartilage abnormalities are being investigated, comprising peptide-based ingredients and injectable hydrogels (Liu et al., 2017). At a 2-year follow-up, pilot research including 28 individuals with osteochondral abnormalities revealed that 70% of lesions were filled utilising tissue and bone nanoscaffold implant (Kon et al., 2011). Further clinical studies have shown mixed outcomes after a 3-year follow-up (Christensen et al., 2016), but further research is being conducted to determine the effectiveness and protection of these scaffolds. Even though nanotechnology has not yet reached broad clinical usage in cartilage regeneration, it has been shown that using nanoparticles as regenerative tissue engineering scaffolds improves chondrocyte adhesion, proliferation, and phenotypic selection (Figure 6) (Parchi, 2013).

## Prospective Concerns

Although recent translational investigation efforts proved nanomedicine’s amazing promise; however, nanomaterials have been linked to cytotoxicity in the lung and brain, oxidative stress, and systemic inflammation in the early stages of research (Polyzois et al., 2012). However, additional research indicates that the outputs of nanoparicles metabolism might potentially promote the health of lung and bone tissue at the microscopic level (Sato and Webster, 2004). The mixture of these two impediments might discourage a large number of medical devices businesses from investing millions of dollars in the capital when sufficient grafts are already available (Nodzo et al., 2015). Since 2008, just 3% of funding for nanotechnology research has gone toward studying its health consequences (Sullivan et al., 2014). Considering these considerations, the significant study will be required to determine the possible toxicity of nanomaterials before they can be extensively employed in therapeutic applications.

Another difficulty is mass producing nanomaterials. According to some experts, the large volume manufacture of materials with a diameter smaller than 03 nm is not repeatable owing to their complicated structural features. Kelly et al. showed that when these nanomaterials are mass-produced on a relatively tiny scale, the size and physical attributes of individual components may vary (Kelly and Dean, 2016). As a result, the elevated, low-cost production paradigm can be impossible to achieve with some nanomaterials without compromising some level of repeatability.

## Future Perspective and Concluding Remarks

Although preliminary research indicates that nanobiomaterials have the potential to be used in orthopaedic applications, more developments are required to attain practical utilization. The objective is to develop bioactive bone repair scaffolds that can partially substitute normal tissues whereas act together with their environment, responding to changes in the environment, and actively directing cellular proceedings to promote faster bone formation, shorter curative times, and speedy return to function. Future work will almost certainly focus on developing improved design techniques that use nanomaterials and other fabrication technologies. Understanding the molecular processes behind cell–nanobiomaterial interactions is crucial. Additionally, confirming the biosafety of nanomaterials and mitigating their effects should be treated carefully. There are challenges regarding the toxicity of nanoparticles formed due to wear and tear. At the nanoscale, metals act differently and display material properties distinct from those at the microscale.

Consequently, traditional implants that have been cured with nanotechnology for precise characteristics are preferable over nanoparticles built as grafts. This eliminates the risk of nanomaterials dispersing and affecting toxicity in the body’s tissues. In light of these concerns, it has been suggested that regulation is necessary. Companies continue to be hesitant to create nanostructured implants and prostheses due to their unproven therapeutic benefits, the potential for toxicity, and high cost (Smith et al., 2018). There are challenges regarding the toxicity of nanoparticles formed due to wear and tear. At the nanoscale, metals act differently and display material properties distinct from those at the microscale.

Although nanotechnology is in its development, it can improve orthopaedic diagnoses, management as well as research. The commercial and service sectors’ performance validates the idea that nanotechnology would play a vital role in therapeutic therapy in the future. Nanotechnology has the capability to reduce the cost of many traditional medicines considerably and to enable a plethora of innovative uses. Nanotechnology enables more precise treatment techniques, resulting in more efficient and durable implants, lower prevention of infection, and enhanced healing of bone and tendon. The potential advantages of nanomedicine are starting to be realized, particularly in orthopaedics, as a result of massive fundamental science research efforts. However, further research is necessary to completely comprehend the safeness and usefulness of this innovative technology.
